# SARS-CoV-2 N Protein Induces Acute Lung Injury in Mice *via* NF-ĸB Activation

**DOI:** 10.3389/fimmu.2021.791753

**Published:** 2021-12-07

**Authors:** Jie Xia, Wenqi Tang, Jiangmei Wang, Dengming Lai, Qi Xu, Ruoqiong Huang, Yaoqin Hu, Xiaojue Gong, Jiajie Fan, Qiang Shu, Jianguo Xu

**Affiliations:** ^1^ The Children’s Hospital of Zhejiang University School of Medicine and National Clinical Research Center for Child Health, Hangzhou, China; ^2^ Hangzhou Medical College of Bioengineering, Hangzhou, China

**Keywords:** SARS-CoV-2, COVID-19, nucleocapsid (N) protein, acute lung injury, NF- kappa B

## Abstract

**Background:**

Infection of SARS-CoV-2 may cause acute respiratory syndrome. It has been reported that SARS-CoV-2 nucleocapsid protein (N-protein) presents early in body fluids during infection. The direct involvement of N-protein in lung injury is poorly understood.

**Methods:**

Recombinant N-protein was pretreated with polymyxin B, a lipopolysaccharide (LPS)-neutralizing agent. C57BL/6, C3H/HeJ (resistant to LPS), and C3H/HeN (control for C3H/HeJ) mice were exposed to N-protein *via* intratracheal administration to examine acute lung injury. *In vitro*, bone marrow–derived macrophages (BMDMs) were cultured with N-protein to study phosphorylation of nuclear factor kappa B (NF-ĸB) p65, macrophage polarization, and expression of proinflammatory cytokines.

**Results:**

N-protein produced acute lung injury in C57BL/6 mice, with elevated protein permeability, total cell count, neutrophil infiltration, and proinflammatory cytokines in the bronchioalveolar lavage. N-protein also induced lung injury in both C3H/HeJ and C3H/HeN mice, indicating that the effect could not be attributed to the LPS contamination. N-protein triggered phosphorylation of NF-ĸB p65 *in vitro*, which was abolished by both N-protein denaturation and treatment with an antibody for N-protein, demonstrating that the effect is N-protein specific. In addition, N-protein promoted M1 macrophage polarization and the expression of proinflammatory cytokines, which was also blocked by N-protein denaturation and antibody for N-protein. Furthermore, N-protein induced NF-ĸB p65 phosphorylation in the lung, while pyrrolidine dithiocarbamate, an NF-ĸB inhibitor, alleviated the effect of N-protein on acute lung injury.

**Conclusions:**

SARS-CoV-2 N-protein itself is toxic and induces acute lung injury in mice. Both N-protein and NF-ĸB pathway may be therapeutic targets for treating multi-organ injuries in Coronavirus disease 2019 (COVID-19).

## Introduction

Coronavirus disease 2019 (COVID-19) has become a global public health emergency since December, 2019. The virus that causes COVID*-*19 is named as SARS*-*CoV*-*2, which belongs to the family of Coronaviruses, including Middle East respiratory syndrome Coronavirus (MERS-CoV) and severe acute respiratory syndrome Coronavirus (SARS-CoV) (1). Infections from MERS-CoV, SARS-CoV, and SARS*-*CoV*-*2 have similar clinical features and may result in acute respiratory distress syndrome (ARDS, acute lung injury) and involvement of other organs (2–4). However, COVID-19 has a mortality rate of 1-2%, which is lower than that of SARS (9.5%) and MERS (34.4%) (5).

ARDS is the most serious complication of COVID-19. It is estimated that the mortality rate of COVID-19-induced ARDS is about 45%, with incidence of ARDS as high as 90% for non-survivors. ARDS is typically characterized by increased proinflammatory cytokines, elevated endothelial permeability, and infiltration of neutrophils and macrophages in the alveolar space. However, there were low expression of type I interferons *(*IFN*-*α and IFN*-*β*)* and high levels of thrombotic mediators in COVID-19-associated ARDS. Severe COVID-19-associated ARDS had higher SARS-CoV-2 viral load, neutrophil count, proinflammatory cytokines, and incidence of thrombosis (6).

The virion envelope of coronaviruses embeds three of the four structural proteins: the spike protein (S), the membrane protein (M), and the envelope protein (E). The fourth, the nucleocapsid protein (N), is located in the interior of the envelope (7). N protein of coronavirus possesses RNA-binding ability and packages RNA genome into a helical nucleocapsid which is important in the initiation of viral replication and infection. Y127A mutation in N protein from mouse hepatitis virus, a virus closely related to SARS-CoV, lost its replication accessory function (8). In addition to viral packaging, N protein plays additional roles during the infection. It interacted with nonstructural protein 3, which is a component of the viral replicase complex, and stimulated the infectivity of mouse hepatitis virus (9). It also bound with the M protein to enhance genome loading and vial assembly (10). In addition, N protein of SARS-CoV induced aggregation of elongation factor 1α and inhibited cytokinesis in human cells (11). Furthermore, the N protein bound with cyclin D, reduced the activity of cyclin-dependent kinase 4/cyclin D complex, and inhibited progression of S phase in mammalian cells (12).

Intranasal administration of SARS-CoV N protein induced inflammatory reactions and pulmonary edema in adult BALB/C mice (13). Gao et al. reported that SARS*-*CoV*-*2 N protein (N-protein) exacerbated lipopolysaccharide (LPS)-induced lung injury by binding to MASP-2, leading to complement activation (14). In a mouse model with overexpression of N-protein mediated by tail vein injection of adenovirus-associated virus-N protein vector, levels of IL-1β and IL-6 were increased in immunohistochemical fluorescence analysis of the lung along with lung inflammation in histology. In addition, N-protein promoted NLRP3 inflammasome activation (15). It is well documented that nuclear factor kappa B (NF-ĸB) is an essential mediator of the inflammatory response and regulates the expression of various pro-inflammatory cytokines involved in acute lung injury (16). However, the role of NF-ĸB in N-protein-induced pathogenesis has not been established. In the present study, we aimed to examine the direct effect of recombinant N-protein on acute lung injury and NF-ĸB activation.

## Materials and Methods

### Preparation and Treatment of Recombinant N-Protein

The construct for hexahistidine (His)-tagged N-protein of SARS*-*CoV*-*2 was kindly provided by Dr. Feng Cong from Guangdong Laboratory Animals Monitoring Institute. The whole sequence of N-protein was prepared *via* PCR, confirmed *via* sequencing, and inserted into pET28a vector for expression as a His-tagged fusion protein. The protein was purified *via* nickle affinity chromatography (BBI Life Sciences, Shanghai, China) and followed by dialysis to remove extra salt and imidazole. To remove the contaminated endotoxin, the recombinant protein was processed with ToxinEraser™ Endotoxin Removal Kit (GenScript, Piscataway, NJ, USA). The final endotoxin concentration was examined by ToxinSensor™ Chromogenic LAL Endotoxin Assay Kit (GenScript). The endotoxin levels in different batches were consistently < 0.1 EU/μg protein (<0.01 ng/μg). The final recombinant protein was aliquoted and stored at -80°C freezer. To neutralize the possible protein-bound LPS in the recombinant protein, N-protein was pretreated with polymyxin B (250 µg/ml, MilliporeSigma, St. Louis) for 1 h at 37°C before all experiments. For denaturation of N-protein, N-protein samples were incubated in a heating block at 80°C for 1 h. To block the effect of N-protein *in vitro*, N-protein (1 mg/ml) was preincubated with an anti-N-protein antibody (Genscript, 1 mg/ml) for overnight at 4°C before the stimulation experiment.

### N-Protein-Induced Lung Injury Model

C57BL/6 mice (6-8 weeks old) were purchased from Shanghai Laboratory Animal Center (Shanghai, China). C3H/HeJ (resistant to LPS) and C3H/HeN (normal response to LPS) mice, 6-8 weeks old, were acquired from Beijing Vital River Laboratory Animal Technology (Beijing, China) and Shanghai Experimental Animal Center (Shanghai, China), respectively. All animals were housed in a temperature-controlled environment at the Zhejiang University Laboratory Animal Center. Protocols for animal research were preapproved by the Institutional Animal Care and Use Committee of Zhejiang University School of Medicine. Mice within each cage were simultaneously randomized into treatment groups in random order. N-protein was pretreated with polymyxin B (250 µg/ml) at 37°C for 1 h for endotoxin neutralization, while bovine serum albumin (BSA) was preincubated at 37°C for 1 h as control. Mice were instilled intratracheally with N-protein (3 μg, 15 μg, and 75 μg per mouse in 50 µL), BSA (75 μg in 50 µL), polymyxin B (50 µl of 250 µg/ml), or phosphate-buffered saline (PBS, 50 µL). To explore the role of NF-ĸB in N-protein-induced lung injury, mice were treated with intraperitoneal injection of NF-ĸB inhibitor pyrrolidine dithiocarbamate (PDTC, 50 mg/kg, MilliporeSigma) 1 h before N-protein treatment. Based on our experience, the optimal duration for N-protein to induce significant acute lung injury was 24 h. Animals were sacrificed at 24 h after N-protein, BSA, polymyxin B, PDTC, or PBS treatment. Lungs and bronchioalveolar lavage (BAL) samples were collected for further analysis.

### Intratracheal Administration of Solution Into Mouse Lung

Intratracheal administration of solution into mice was modified from Helms et al. (17). Mice were anesthetized with phenobarbital (50 mg/kg) *via* intraperitoneal injection. The fur covering the tracheal region was shaved with an electric razor. A layer of hair removal cream was applied to the shaved area and cleaned with a damp towel after 3 minutes of application. The surgical area was wiped with alcohol pads. The anesthetized mice were positioned onto an angled restraining stand. A small skin incision was made in the tracheal region. The anterior tracheal muscles were dissected with blunt incision to visualize and access the tracheal rings. Fifty µl of solution was administered into trachea using a 3/10 mL insulin syringe with 30 gauge x ½ inch needle. The solution was injected slowly with the needle bevel up and parallel with the trachea. To confirm the success of instillation, air was introduced through the syringe and expansion of lung was observed. The needle was removed from the trachea. Mice were then allowed to recover in room air for 30 min and returned to the cages with free access to food and water.

### Inflammatory Cell Counts, Protein, and Cytokines in BAL

To obtain BAL cells from mice, the tracheas were canulated and the lungs were lavaged three times with 0.5 ml of cold PBS. Total white blood cell counts in the BAL were directly examined using a hemocytometer. BAL was then labeled with FITC-conjugated anti-mouse Ly-6G (Gr-1) antibody (Thermo Fisher Scientific, Waltham, MA) and analyzed for the percentage of neutrophils *via* flow cytometry. Neutrophil counts in the BAL were calculated by multiplying the percentage of neutrophils by the total number of white blood cells. BAL samples were then centrifuged at 400 x g for 5 min at 4°C to collect supernatant. Protein concentration in BAL supernatant, which is an index of capillary leakage during lung inflammation, was determined using a BCA Protein Assay Kit (Thermo Fisher Scientific). Cytokine levels of interleukin (IL)-1β, IL-6, tumor necrosis factor (TNF)-α, and interferon (IFN)-γ in the BAL were examined by ELISA (R&D Systems, Minneapolis, MN) per manufacturer’s protocol.

### Histopathology

BAL collection and lung tissue pathology were performed on separate mice. To harvest the lungs for histological analysis, the tracheas were cannulated with a 18G needle and the lungs inflated with 0.6 ml of chilled 4% paraformaldehyde. After fixation for 24 h, lung tissue was embedded in paraffin wax and sectioned at 5 μm in thickness. Lung sections were then stained with hematoxylin and eosin (H&E) to assess cellular and morphological structures. Images of lung sections were taken with an Olympus VS120 microscope (Shinjuku, Tokyo, Japan).

### Flow Cytometry for Alveolar Macrophages From *In Vivo* Studies

To determine macrophage recruitment and phenotype in BAL, cells were labelled with the following surface markers: PE anti-mouse F4/80 (BioLegend, San Diego, CA), BV650 anti-mouse CD86 (BD, Franklin Lakes, NJ), PE-cy7 anti-mouse iNOS (Thermo Fisher Scientific), APC anti-mouse CD206 (Thermo Fisher Scientific) for 20 min at 4°C. Cells were then washed twice with cold flow cytometry buffer (PBS, 0.5% BSA) and incubated for 30 min at 4°C. Cells were subsequently washed and resuspended with cold flow cytometry buffer for analysis. Isotype-matched control antibodies were used to distinguish the cut-off between negative and positive fluorescent populations. Data were collected by a BD LSRFortessa™ flow cytometer and analyzed using FlowJo V10 software.

### Isolation, Treatment, and Phenotype Analysis of Mouse Bone Marrow-Derived Macrophages (BMDMs)

For BMDMs, C57BL/6 mice (6-8 weeks old) were sacrificed *via* cervical dislocation to harvest femurs and tibias. Bone marrow was flushed out of the ends of the bones by inserting a 25-gauge needle attached to a 10 ml syringe filled with PBS, until the color of the bone turned from red to white. Bone marrow was further dispersed by passing through an 19-gauge needle attached to a 10 ml syringe filled with PBS. The cell suspension was collected and centrifugated at 250 g for 5 min at room temperature. Red blood cells in the pellet were lysed by incubating with 1x lysis buffer (10 mM KHCO3, 155 mM NH4Cl, 0.1 mM EDTA) for 3 min. Cells were pelleted again, washed with PBS, and cultured in medium containing DMEM supplemented with GM-CSF (20 ng/ml, PeproTech, Rock Hill, NJ), 10% fetal bovine serum (Biological Industries, Cromwell, CT), and 1% penicillin plus streptomycin at 37°C with 5% CO2 overnight. Non-adherent cells were harvested and seeded on 6-well culture plates (2 x 10^6^ cells/well). Fresh culture medium was fed every 3 days. Adherent BMDMs were harvested with lidocaine/EDTA (0.4% lidocaine, 5 µM EDTA) after 7 days and quantitated for further experiments. BMDMs (5 x 10^5^ cells) were cultured in a standard 12-well plate and stimulated with N-protein (5 µg/ml), LPS (10 ng/ml), or PBS for different time periods. Then, BMDMs were harvested for Western blot, qRT-PCR, and phenotype analyses. To determine phenotypes of BMDMs, cells were labelled with the following surface markers: PE anti-mouse F4/80 and BV650 anti-mouse CD86 for 20 min at 4°C. BMDMs were then washed twice with cold flow cytometry buffer (PBS, 0.5% BSA) and incubated for 30 min at 4°C. Then, cells were washed and resuspended with cold flow cytometry buffer for analysis. Data were collected by a BD LSRFortessa™ flow cytometer and analyzed using FlowJo V10 software.

### Western Blot Analysis

Whole-cell lysates of BMDMs were prepared in lysis buffer (10 mM Tris-HCl, pH 7.4, 1 mM EDTA, 150 mM NaCl, 0.5% Nonidet P-40, 1 mM Na_3_VO_4_, and 1 mM PMSF). The total protein concentration was determined using a BCA protein assay kit, exhibiting strong absorbance at 562 nm. Equal amount of protein extracts (30 μg) or N-protein (0.5 μg) were run on 12% sodium dodecyl sulfate-polyacrylamide gel electrophoresis (SDS-PAGE), transferred to polyvinylidenefluoride membranes (Millipore, Billerica, MA), blocked with 5% non-fat milk in tris-buffered saline with Tween-20 (TBST), and probed with the primary antibodies to N-protein (Genscript), phosphor-NF-ĸB p65 (#3033, Cell Signaling Technology, Danvers, MA), and NF-ĸB p65 (#8242, Cell Signaling Technology). After washing with TBST, membranes were incubated with specific secondary antibodies conjugated to horseradish peroxidase. The binding was detected *via* enzyme-linked chemiluminescence using the EZ-ECL kit (Biological Industries, Kibbutz Beit-Haemek, Israel).

### Quantitative Real-Time PCR (qRT-PCR)

Total RNA was isolated from the cells with the use of Trizol reagent (Thermo Fisher Scientific). The RNA concentration was determined by a Nanodrop Spectrophotometer (ND-2000; Thermo Fisher Scientific). RNA (1 µg) was reversely transcribed to cDNA using the Superscript III First-strand kit (Thermo Fisher Scientific). The resulting cDNA was amplified by Power SYBR Green RT-PCR reagent kit (Takara Bio, Kusatsu, Japan). mRNA values were normalized for differences in sample concentration to the levels of β-actin mRNA and expressed as relative fold change compared with the reference of control samples. PCR primer sequences were listed as follows: IL-1β forward 5’GAAATGCCACCTTTTGACAGTG3’, reverse 5’TGGATGCTCTCATCAGGACAG3’; IL-6 forward 5′CTGCAAGAGACTTCCATCCAG3′, reverse 5′AGTGGTATAGACAGGTCTGTTGG3′; TNF-α forward 5’CAGGCGGTGCCTATGTCTC3’, reverse 5’CGATCACCCCGAAGTTCAGTAG3’; inducible nitric oxide synthase (iNOS) forward 5’CAGGCTGGAAGCTGTAACAAAG3’, reverse 5’GAAGTCATGTTTGCCGTCACTC3’; YM-1 forward 5’CAGGTCTGGCAATTCTTCTGAA3’, reverse 5’GTCTTGCTCATGTGTGTAAGTGA3’; CD206 forward, 5’CTCTGTTCAGCTATTGGACGC3’, reverse 5’TGGCACTCCCAAACATAATTTGA3’; β-actin forward 5’CGTTGACATCCGTAAAGACC3’, reverse 5’AACAGTCCGCCTAGAAGCAC3’.

### Statistical Methods

Data are presented as mean ± standard deviation of the mean (SD). Student’s t test, one-way analysis of variance (ANOVA) with Tukey’s *post hoc* analysis, or two-way AVOVA with Tukey’s *post hoc* analysis (**Figure 4** only) was performed for parametric data with n ≥ 8. The Mann-Whitney test or Kruskal-Wallis test with Dunn’s *post hoc* analysis was performed for small sample size (n < 8) and non-parametric data with n ≥ 8. Data were tested for normality by using the D’Agostino-Pearson Omnibus normality test, the Shapiro-Wilk normality test, and Kolmogorov-Smirnov test with Dallal-Wilkinson-Lilliefor corrected P value. Statistical analysis was carried out using the GraphPad Prism 7. Results were considered significant if p < 0.05.

## Results

### Recombinant SARS-CoV-2 N-Protein Prompts Pulmonary Inflammation

**Figure 1:**
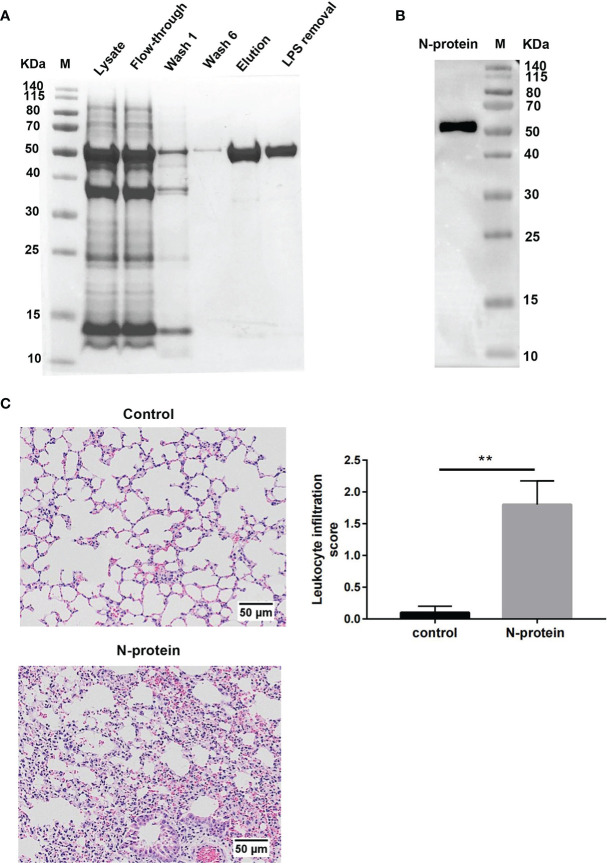
Effect of N-protein on lung inflammation. **(A)** His-tagged SARS-CoV-2 N-protein was purified on a Ni-NTA column. Samples at various stage of purification were run on 12% SDS-PAGE gel and stained with Coomassie blue. M: molecular weight marker. LPS removal: LPS removed *via* a commercial kit after purification. **(B)** Purified N-protein was separated on 12% SDS-PAGE gel and examined for N-protein identity *via* Western blot analysis. M: molecular weight marker. **(C)** N-protein was pretreated with polymyxin B (250 µg/ml) for 1 h at 37°C. C57BL/6 mice (6-8 weeks old) were treated with PBS or N-protein (75 µg/mouse) intratracheally. Lung samples were harvested at 24 h after N-protein insult. Tissue sections were stained with H&E and viewed at x100 magnification. Leukocyte infiltration was scored semi-quantitatively from 0 (normal tissue) to 3 (intensive filtration) from 6 randomly selected fields in three tissue sections per mouse from four mice. Data are presented as mean ± SD, n = 4. **p < 0.01.

To study the effects of N-protein on lung injury, recombinant N-protein was prepared as a His-tagged fusion protein (**Figure 1A**). The molecular size of the protein was approximately 50 kDa. Coomassie blue staining showed greater than 95% purity of affinity-purified N-protein (**Figure 1A**). The endotoxin level was <0.1 EU/μg protein (<0.01 ng/μg) after processed with an endotoxin removal kit. The identity of the purified protein was confirmed by Western blot analysis with an anti-N-protein antibody (**Figure 1B**). To address the possibility that protein-bound LPS might escape detection, N-protein was pretreated with polymyxin B (250 µg/ml) at 37 °C for 1 h for all *in vivo* and *in vitro* experiments. Incubation of N-protein with polymyxin B did not result in protein aggregation (**Supplemental Figure 1**). To determine the effects of N-protein on pulmonary inflammation, C57BL/6 mice were treated intratracheally with N-protein (75 µg/mouse) or PBS. Treatment of N-protein resulted in an acute inflammatory response, with thickened alveolar septa and increased cellularity compared to control at 24 h (**Figure 1C**). There was a significant increase in the leukocyte infiltration score in the lung of N-protein group compared with the control. Similar doses of proinflammatory stimuli, such as HMG-1 and SARS S protein, were reported to induce lung inflammation in animal studies (18, 19).

### Effects of N-Protein on Acute Lung Injury Are Dose-Dependent

**Figure 2:**
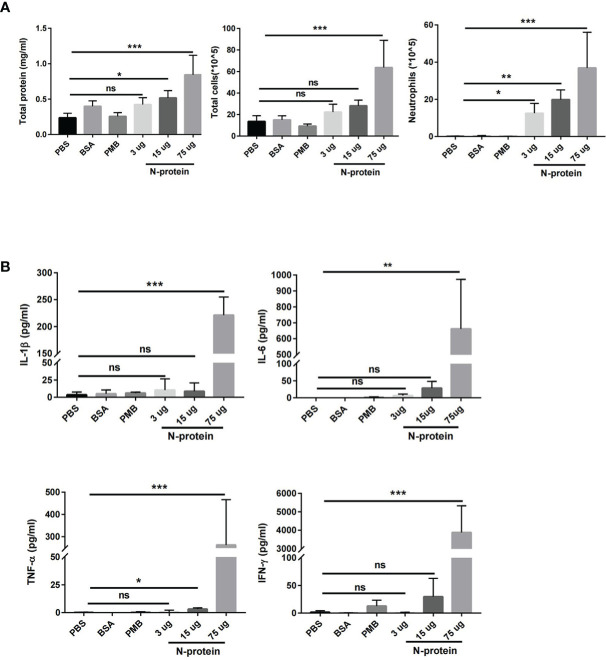
Dose-dependent effect of N-protein on lung injury. C57BL/6 mice (6-8 weeks old) were divided into 6 groups: control, bovine serum albumin (BSA, 75 µg in 50 µl), polymyxin B (PMB, 250 µg/ml, 50 µl), N-protein (3 µg in 50 µl), N-protein (15 µg in 50 µl), and N-protein (75 µg in 50 µl). **(A)** Total protein, total cells, and neutrophils in the BAL were examined at 24 h after N-protein insult to examine inflammatory response. **(B)** IL-1β, IL-6, TNF-α, and IFN-γ levels in the BAL were determined *via* ELISA. Data are presented as mean ± SD, n = 8-10. *p < 0.05, **p < 0.01, ***p < 0.001. ns, not significant.

C57BL/6J mice were treated with N-protein at three dose levels intratracheally (3, 15, and 75 µg/mouse in 50 µl). BAL fluids were harvested at 24 h after N-protein exposure and analyzed for pulmonary endothelial permeability, cellular infiltration, and concentrations of proinflammatory cytokines. Pulmonary endothelial permeability, as determined by total protein levels in the BAL, was significantly elevated in the medium dose (15 µg/mouse) and high dose (75 µg/mouse) groups compared with PBS control (**Figure 2A**). High dose of N-protein also increased the total number of cells in the BAL, while neutrophils were raised in all 3 dose groups compared with control. In addition, the concentrations of proinflammatory cytokines IL-1β, IL-6, TNF-α, and IFN-γ were significantly higher in the high dose group than the control (**Figure 2B**). Medium dose of N-protein only raised the TNF-α level, while low dose had no effect on all four cytokines. In contrast, intratracheal administration of bovine serum albumin (75 µg/mouse) or polymyxin B (50 µl of 250 µg/ml) did not result in any significant change in the above parameters for lung injury.

### N-Protein Promotes M1 Macrophage Polarization *In Vivo*

**Figure 3:**
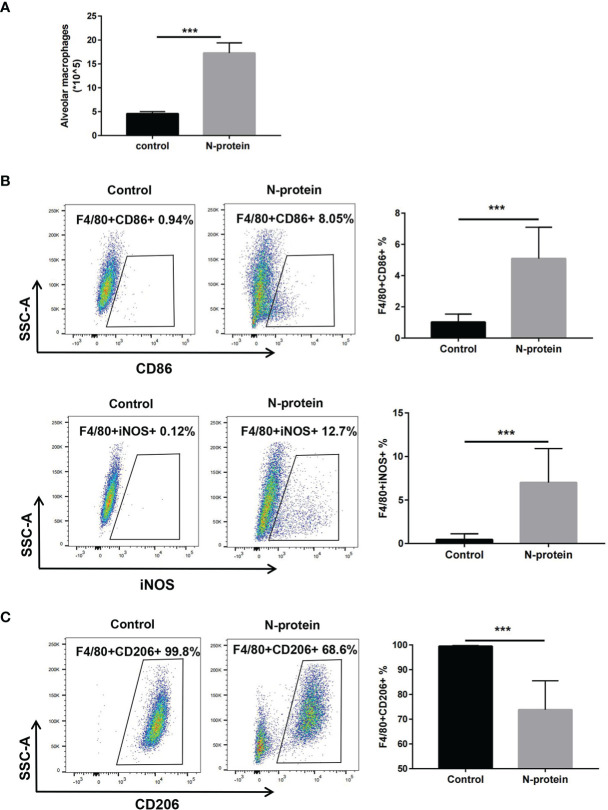
M1 polarization of alveolar macrophages after N-protein treatment *in vivo*. C57BL/6 mice were treated with PBS or N-protein (75 µg/mouse in 50 µl) intratracheally. Total cells in the BAL were harvested at 24 h and counted using a hemocytometer. **(A)** Alveolar macrophages in the BAL were identified as F4/80+ in flow cytometry and quantitated by the percentage of the positive cell population and the total cells in the BAL. Dot plots showed the gating scheme for determining the frequency of F4/80+CD86+ (M1) **(B)**, F4/80+iNOS+ (M1) **(B)**, and F4/80+CD206+ (M2) **(C)** cells in flow cytometry. The numbers in the dot plots represent the percent of the population within the specific gate. Percentages of the M1/M2 populations were summarized in the panels at right. Data are presented as mean ± SD, n = 6-8. ***p < 0.001.

To study the effects of N-protein on macrophage polarization, mice were subjected to N-protein (75 µg/mouse) or PBS intratracheally. BAL was harvested at 24 h and analyzed phenotypically *via* flow cytometry (**Supplemental Figure 2** for gating strategy). Flow cytometry analysis demonstrated that N-protein increased the number of macrophages (F4/80+) in the BAL compared with the control (**Figure 3A**). The results also showed that N-protein significantly elevated the expression of F4/80+CD86+ and F4/80+iNOS+ cells, favoring the orientation toward M1 macrophages (**Figure 3B**). In addition, N-protein decreased the levels of F4/80+CD206+ cells, a marker for M2 macrophages (**Figure 3C**).

### N-Protein Induces Lung Injury in Mice With TLR4 Mutation

**Figure 4:**
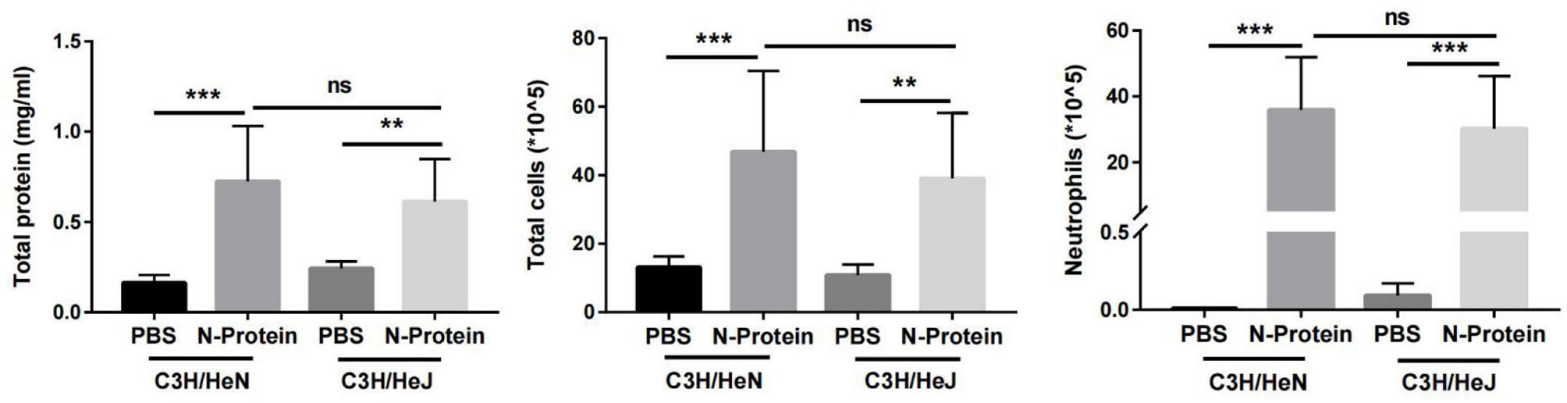
Effect of N-protein on lung injury in C3H/HeJ (TLR4 mutation) and C3H/HeN (control) mice. C3H/HeJ and C3H/HeN mice were treated with PBS or N-protein (75 µg/mouse in 50 µl) intratracheally. Total protein, total cells, and neutrophils in the BAL were examined at 24 h after N-protein insult to examine inflammatory response. Data are presented as mean ± SD, n = 8. Two-way AVOVA with Tukey’s *post hoc* analysis, **p < 0.01, ***p < 0.001. ns, not significant.

To substantiate that N-protein-induced lung injury is not the result of LPS contamination, N-protein-induced lung injury was compared between C3H/HeJ (TLR4 mutation) and C3H/HeN (control) mice. Previous studies have reported that mice with TLR4 mutation are resistant to LPS-induced lung injury (20). Mice were administered with N-protein (75 µg/mouse) or PBS, and then examined for lung injury at 24 h. TLR-4 mutation did not alter the effects of N-protein on total protein concentration, total cell count, and neutrophil infiltration in the BAL compared with control mice, indicating N-protein is responsible for the lung injury (**Figure 4**).

### N-Protein Activates NF-ĸB Signaling Pathway *In Vitro*

**Figure 5:**
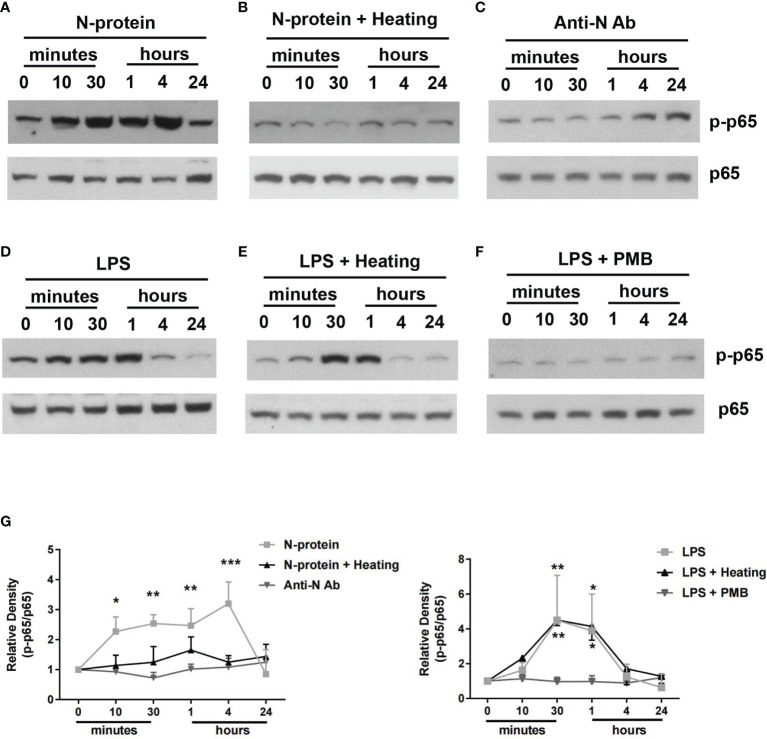
NF-ĸB activation by N-protein. BMDMs were stimulated with N-protein (5 µg/ml) or LPS (10 ng/ml) for the indicated times. Western blot was performed using antibodies specific to phospho-NF-ĸB p65 and total NF-ĸB p65. N-protein for all groups was pretreated with polymyxin B (250 µg/ml) for 1 h at 37°C before experiment. **(A)** N-protein was added to the culture medium directly. **(B)** N-protein was denatured *via* heat inactivation (Heating) (80°C, 1 h). **(C)** N-protein was treated with an anti-N-protein antibody (Anti-N Ab) prior exposure to BMDMs. **(D)** LPS (10 ng/ml) induced NF-ĸB activation in BMDMs at different time points. **(E)** LPS was heated at 80°C for 1 h before stimulation. **(F)** LPS was pretreated with polymyxin B (250 µg/ml) for 1 h at 37°C before stimulation. **(G)** The band density of phospho-NF-ĸB p65 was normalized to that of total NF-ĸB p65 and expressed as relative fold change compared with time 0. Data are presented as mean ± SD, n = 3. *p < 0.05, **p < 0.01, ***p < 0.001 *versus* time 0.

To examine the signal transduction pathways involved, BMDMs were exposed to polymyxin B-treated N-protein (5 µg/ml) and activation of NF-ĸB was examined by Western blot, using a phospho-specific p65 antibody. N-protein triggered phosphorylation of NF-ĸB p65, beginning at 10 min after the exposure, reaching a peak between 30 min to 4 h, and then returning to the baseline at 24 h (**Figures 5A, G**). In addition, the NF-ĸB activation was lost after N-protein denaturation *via* heat inactivation (80°C, 1 h) (**Figure 5B**). Furthermore, incubation with an anti-N-protein antibody blocked the effect of N-protein on NF-ĸB p65 phosphorylation (**Figure 5C**). As a control, LPS (10 ng/ml) had a relatively short duration for NF-ĸB activation (**Figures 5D, G**), with a peak between 30 min to 1 h. Additionally, LPS was not inactivated at the temperature and duration applied to denature N-protein as the p65 phosphorylation pattern was unaltered (**Figure 5E**). Moreover, LPS induced NF-ĸB signaling was efficiently inhibited by polymyxin B treatment (**Figure 5F**).

### N-Protein Enhances M1 Macrophage Polarization and the Expression of Proinflammatory Cytokines *In Vitro*

**Figure 6:**
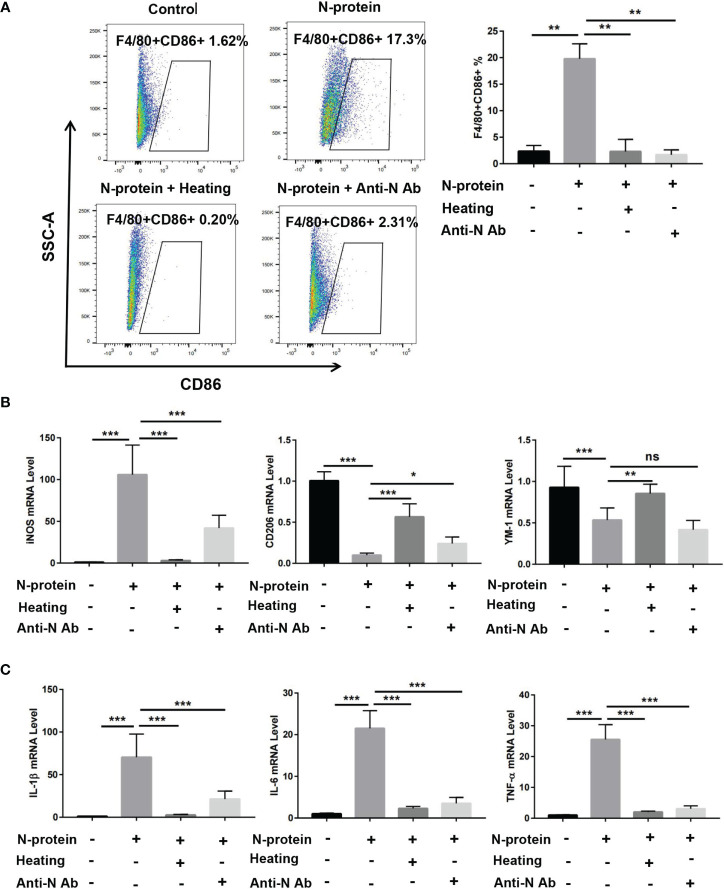
Modulation of macrophage polarization and production of proinflammatory cytokines *in vitro* by N-protein. BMDMs were divided into 4 groups: control, N-protein (5 µg/ml), N-protein with heat denaturation (Heating), pretreatment of N-protein with an anti-N-protein antibody (Anti-N Ab). Cells were harvested at 24 h after treatment. **(A)** Left, dot plots showed the gating scheme for determining the frequency of F4/80+CD86+ (M1). Right, the percentage of F4/80+CD86+ macrophages were quantified. **(B)** mRNA levels of macrophage phenotype markers iNOS, CD206, and YM-1 were assayed *via* qRT-PCR. **(C)** mRNA levels of pro-inflammatory cytokines IL-1β, IL-6, and TNF-α were analyzed *via* qRT-PCR. Data are presented as mean ± SD, n = 6–9. *p < 0.05, **p < 0.01, ***p < 0.001. ns, not significant.

Prior studies have demonstrated that activation of NF-κB promotes M1 macrophage polarization, resulting in proinflammatory responses (21). To study whether N-protein modulates macrophage phenotypes *in vitro*, BMDMs were cultured with polymyxin B-treated-N-protein (5 µg/ml) or PBS for 24 h. Flow cytometry results showed that N-protein enhanced the level of M1 macrophages (F4/80+CD86+) compared with the control (**Figure 6A**). N-protein also elevated the mRNA expression of M1 macrophage marker iNOS while simultaneously decreased the levels of M2 markers CD206 and YM-1 (**Figure 6B**). The effect of N-protein on macrophage polarization was abolished by N-protein denaturation *via* heating. Meanwhile, pretreatment with N-protein antibody displayed similar effects with the exception of elevating YM-1 expression (**Figures 6A, B**). In addition, mRNA levels of proinflammatory IL-1β, IL-6, TNF-α were raised by N-protein treatment (**Figure 6C**). Conversely, N-protein denaturation and N-protein antibody blocked the impacts of N-protein on proinflammatory cytokines.

### N-Protein Activates NF-ĸB Signaling *In Vivo*

**Figure 7:**
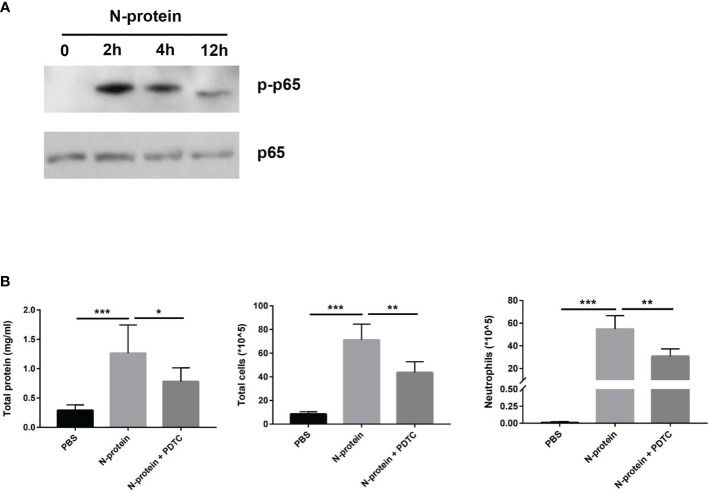
Effect of N-protein on NF-ĸB activation *in vivo*. **(A)** C57BL/6 mice were treated with N-protein (75 µg/mouse in 50 µl) intratracheally for 0, 2, 4, or 12 h. BAL cells were harvested at different time points and examined for phosphor-NF-ĸB p65 and total NF-ĸB p65 *via* Western blot. **(B)** Mice were treated with PBS, N-protein (75 µg/mouse), or N-protein + NF-κB inhibitor PDTC (50 mg/kg, intraperitoneal injection 1 h before N-protein treatment). Total protein, total cells, and neutrophils in the BAL were examined at 24 h after N-protein insult to examine inflammatory response. Data are presented as mean ± SD, n = 7-9. *p < 0.05, **p < 0.01, *** p < 0.001.

To assess the effect of N-protein on NF-ĸB activation in the lungs, C57BL/6 mice were treated with N-protein intratracheally (75 µg/mouse in 50 µl) for 0 (immediate before treatment), 2, 4, or 12 h. Levels of phospho-specific NF-ĸB p65 were measured by Western blot of lysates from BAL cells (**Figure 7A**). NF-ĸB p65 phosphorylation was undetectable at time 0, dramatically elevated at 2 h after the exposure, and gradually decreased at 4 and 12 h. To establish that NF-κB mediates N-protein-induced lung injury, mice were treated with PBS, N-protein (75 µg/mouse), or N-protein + NF-κB inhibitor PDTC (50 mg/kg). PTDC alleviated the effects of N-protein on total protein concentration, total cell count, and neutrophil infiltration in the BAL at 24 h, indicating that NF-κB activation is at least partially responsible for the lung injury (**Figure 7B**).

## Discussion

The present study reveals that SARS-CoV-2 N-protein causes acute lung injury in mice *via* activation of NF-ĸB. This conclusion is substantiated by the following findings. (1) Recombinant N-protein induced dose-dependent effects on acute lung injury in mice. (2) N-protein enhanced M1 macrophage polarization *in vivo*. (3) The effect of N-protein was not due to LPS contamination since mice with TLR4 mutation had comparable response to N-protein compared with control. (4) N-protein activated NF-ĸB p65 phosphorylation which was abolished by both N-protein denaturation and an antibody to N-protein. (5) Macrophages treated with N-protein displayed features of NF-ĸB activation with elevated levels of proinflammatory cytokines and M1 macrophage polarization. (7) N-protein-induced lung injury was alleviated by an NF-ĸB inhibitor.

Some literature suggests that many of the reported effects of recombinant proteins are a result of contamination of LPS (22). To minimize LPS contamination in our recombinant N-protein, the protein samples eluted from column were treated with an endotoxin removal system. The resulting protein had LPS level of <0.01 ng/μg protein by the limulus amebocyte assay. In our *in vivo* experiments, the possible contaminated LPS dose received per mouse was <0.75 ng (75 μg of N-protein with LPS level of <0.01 ng/μg). Most of the LPS-induced lung injury models in the literature administer a dose of 1-10 mg/kg to mice (23). This indicates that the LPS dose to induce lung injury in mouse is at least in the μg scale, which is more than 1000-fold higher than the level of possibly contaminated LPS in the present study. In addition, N-protein was pretreated with polymyxin B (250 µg/ml) in all of our experiments. It was reported that polymyxin B (25 µg/ml) could efficiently abolish the effect of LPS (10 ng/ml), which was estimated to be more than 1,000-fold higher than the possible contamination in the recombinant protein (24). Furthermore, N-protein-induced lung injury was shown in mice with TLR4 (LPS receptor) mutation. In summary, these findings demonstrate that N-protein-induced lung injury in the present study is not due to contamination by LPS.

Recently, Pan et al. reported that virus-mediated overexpression of N-protein, which was introduced *via* systemic administration, enhanced NLRP3 inflammasome activation and induced hyperinflammation of the lung (15). Our research offers several unique findings. First, recombinant N-protein was administered to the lung intratracheally to observe the direct effect of N-protein. Secondly, in addition to the general features of lung inflammation, we studied the hallmarks of acute lung injury. The hallmarks included elevated pulmonary microvascular permeability, as reflected by increased protein levels in the BAL, as well as neutrophil infiltration. Third, we observed that N-protein activated NF-ĸB pathway.

In the present study, mice were treated with 75 µg of N-protein intratracheally to induce acute lung injury. In clinical practice, the typical drug dose given *via* endotracheal route is 2 to 2.5 times the recommended intravenous dose (25). This indicates that approximately 30 µg of the administered N-protein reached the circulation. It has been reported that the serum N-protein concentration was closely correlated to disease severity and had a peak value from 1 ng/ml to 2.7 µg/ml (26, 27). The concentration of N-protein in BAL of COVID-19 patients has not been documented in the literature. Although it is difficult to translate the N-protein dose from mice to humans, the present study may provide important insights into the pathophysiology of COVID-19.

In addition to N protein, other proteins of coronavirus have also been reported to cause lung injury. Kuba et al. first documented that S protein of SARS-CoV exacerbated acid-induced acute lung in mice, which was alleviated by inhibiting the renin-angiotensin pathway (19). S protein of SARS-CoV-2 was shown to aggravate TLR agonist polyinosinic-polycytidylic acid-induced lung inflammation (28). Cao et al. found that S protein of SARS-CoV-2 alone produced lung injury by activating macrophages (29). Another study reported that SARS-CoV-2 S protein induced lung injury *via* impaired endothelial function by downregulation of angiotensin-converting enzyme 2 (ACE 2) (30). Furthermore, overexpression of SARS-CoV-2 E protein induced cell death in multiple cell lines and elicited inflammatory response in macrophages. Intravenous administration of purified E protein caused acute lung injury in mice (31).

The pathogenesis of SARS-CoV-2 is very complex and remains to be understood. Among the three surface proteins of SARS-CoV-2, the S protein is crucial to mediate the viral entry *via* the respiratory tract and target ACE2 on alveolar epithelial cells, vascular endothelial cells, and alveolar macrophages (32). After ACE2 binding, S protein is cleaved by TMPRSS2, which leads to the fusion between the virus envelope and cell membrane (33). In the meantime, the nucleocapsid containing viral genome is released into the host cytosol. Then, the viral genome is translated into proteins such as RNA-dependent RNA polymerase (nsp12), facilitating RNA replication (34). The viral RNA is further packaged with the basic N-protein to form a helical capsid to prevent the degradation by RNase (35). The viral replication leads to dysregulated activation of macrophages in the lung and trigger the massive release of proinflammatory cytokines such as IL-1, IL-6, IL-8, and TNF-α, resulting in epithelial damage, endothelial injury, and elevated vascular permeability. The activated macrophages also release chemokines such as C-chemokine ligand 2 (CCL2), CCL3, and CCL5, resulting in further accumulation of monocyte-derived macrophages (36). Our finding that N-protein itself is a direct cause of acute lung injury is an addition to the expanding body of COVID-19 literature.

The NF-ĸB pathway is responsive to stress stimuli, such as cytokines, pathogenic infection, and environmental stress. It activates downstream effectors such as transcription factors to enhance the production of proinflammatory cytokines (37). Several studies have documented the role of NF-ĸB pathway in Coronavirus infection. Huang et al. reported that when induced pluripotent stem cell-derived alveolar type 2 cells were infected with SARS-CoV-2, NF-ĸB pathway was activated with upregulation NF-ĸB target genes such as IL-6, IL-8, GM-CSF, and VEGF (38). In SARS-CoV sensitive Vero E6 cells, NF-ĸB pathway was stimulated by SARS-CoV N-protein in a dose-dependent manner (39). It has been postulated that ACE-2-NF-κB pathway activation might be responsible for high risk of severe illness from COVID-19 in diabetic and elderly individuals (40). The molecular mechanism underlying N-protein-induced NF-ĸB activation warrants further investigation.

In conclusion, our results demonstrate that SARS-CoV-2 N-protein itself promotes acute lung injury in mice. N-protein activates NF-ĸB pathway and enhances the expression of proinflammatory cytokines. Therapeutic interventions targeting at N-protein and/or NF-ĸB pathway may alleviate cytokine storm and ARDS in COVID-19.

## Data Availability Statement

The original contributions presented in the study are included in the article/**Supplementary Material**. Further inquiries can be directed to the corresponding authors.

## Ethics Statement

The animal study was reviewed and approved by Institutional Animal Care and Use Committee of Zhejiang University School of Medicine.

## Author Contributions

Conception and design: JX, QS, and JGX. Acquisition of Data: JX, WT, JW, DL, QX, RH, YH, XG, JF, QS, and JGX. Analysis and interpretation: JX, WT, JW, DL, QX, RH, YH, XG, JF, QS, and JGX. All authors contributed to drafting the manuscript of important intellectual content and final approval of the manuscript.

## Funding

This work was supported by the Natural Science Foundation of Zhejiang Province (Grant No. LQY19H190001), the Health Commission of Zhejiang Province (2021KY188), the Zhejiang Provincial Program for the Cultivation of High-level Innovative Health Talents (Grant No. 2016-6), and the National Scientific Foundation of China (Grant No. 81671956, 81772122, 82070074).

## Conflict of Interest

The authors declare that the research was conducted in the absence of any commercial or financial relationships that could be construed as a potential conflict of interest.

## Publisher’s Note

All claims expressed in this article are solely those of the authors and do not necessarily represent those of their affiliated organizations, or those of the publisher, the editors and the reviewers. Any product that may be evaluated in this article, or claim that may be made by its manufacturer, is not guaranteed or endorsed by the publisher.

## Abbreviations

ARDS, acute respiratory distress syndrome; LPS, lipopolysaccharide; N-protein, SARS*-*CoV*-*2 N protein; NF-ĸB, nuclear factor kappa B*;* BMDMs, bone marrow-derived macrophages; COVID-19, coronavirus disease 2019; MERS, Middle East respiratory syndrome Coronavirus; SARS, severe acute respiratory syndrome Coronavirus; S, spike protein; M, membrane, and E, envelope; SDS-PAGE, sulfate-polyacrylamide gel electrophoresis; TBST, tris-buffered saline with Tween-20; qRT-PCR, quantitative real-time polymerase chain reaction; iNOS, inducible nitric oxide synthase; His, hexahistidine; PMB, polymyxin B; BAL, bronchoalveolar lavage; H&E, hematoxylin and eosin; IFN, Interferon; IL, Interleukin; TNF, tumor necrosis factor; BSA, bovine serum albumin; PBS, phosphate-buffered saline; ACE2, angiotensin-converting enzyme 2.
