# Supplementary material for: SARS-CoV-2 N Protein Induces Acute Lung Injury in Mice via NF-ĸB Activation
Source: Front Immunol. 2021 Dec 7;12:791753. doi: 10.3389/fimmu.2021.791753 (PMC8688532; doi:10.3389/fimmu.2021.791753)
Supplement: Supplementary file 1 [file DataSheet_1.docx]

**Online Data Supplement for**

**SARS-CoV-2 N protein induces acute lung injury in mice via NF-**ĸ**B activation**

Jie Xia^1^, Wenqi Tang^1^, Jiangmei Wang^1^, Dengming Lai^1^, Qi Xu^2^, Ruoqiong Huang^1^, Yaoqin Hu^1^, Xiaojue Gong^1^, Jiajie Fan^1^, Qiang Shu^1,^*, Jianguo Xu^1,^*

^1^The Children’s Hospital of Zhejiang University School of Medicine and National Clinical Research Center for Child Health, 3333 Binsheng Road, Hangzhou, Zhejiang, 310052, China

^2^Hangzhou Medical College, 182 Tianmu District, Hangzhou, Zhejiang, 310025, China

**Supplemental figures**


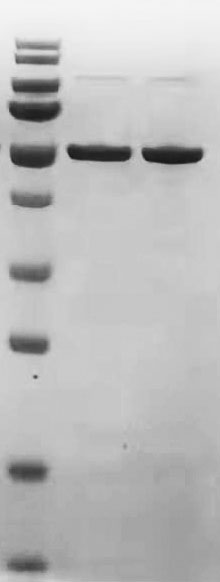


**M**

**1**

**KDa**

**70**

**50**

**140**

**115**

**80**

**40**

**30**

**25**

**15**

**10**

**2**

Supplemental Figure 1. N-protein did not form aggregates after incubation with polymyxin B. N-protein (1.5 µg) before and after incubation with polymyxin B for 1 h at 37 ^o^C was analyzed by native gel electrophoresis (12% polyacrylamide). The sample and running buffers were prepared in the absence of denaturing or reducing agents. The gel was stained with Coomassie blue. Lane 1, before incubation; Lane 2, after incubation.


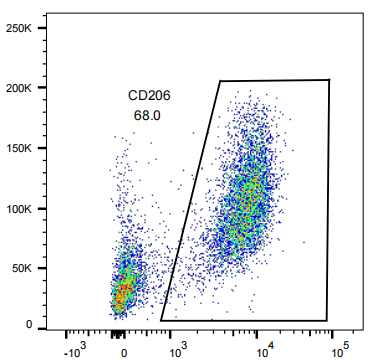

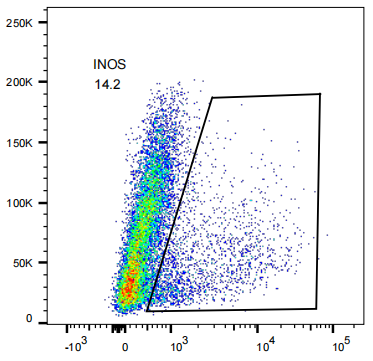

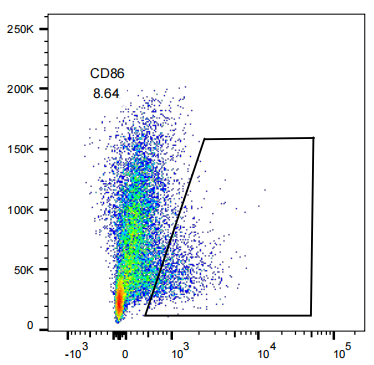

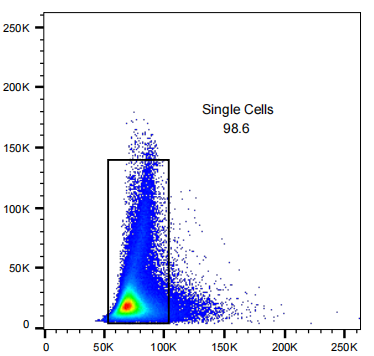

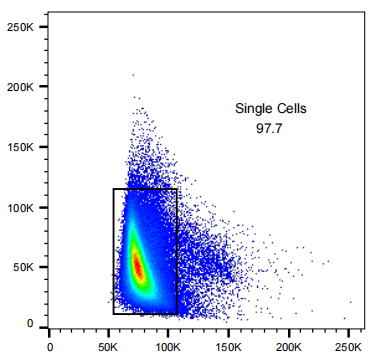

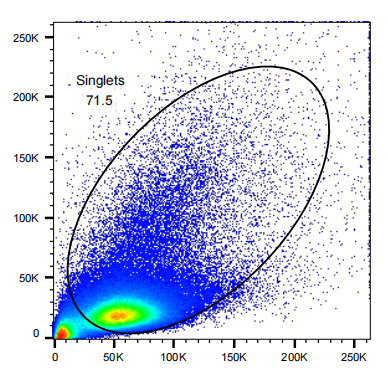

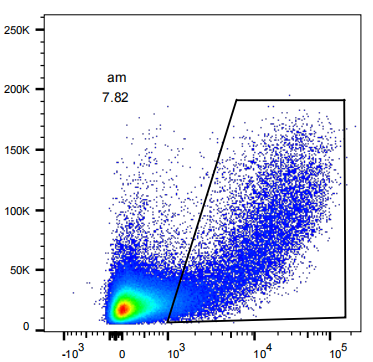


**SSC-A**

**FSC-A**

**FSC-W**

**FSC-H**

**SSC-W**

**SSC-H**

**SSC-A**

**F4/80**

**SSC-A**

**iNOS**

**SSC-A**

**CD86**

**SSC-A**

**CD206**

**F4/80+CD86+**

**F4/80+iNOS+**

**F4/80+CD206+**

Supplemental Figure 2. Gating strategy for M1 and M2 macrophages in the BAL. BAL cells were gated on the basis of their forward and side scatter (FSC/SSC). Macrophages were identified as F4/80+. M1 macrophages were gated as F4/80+CD86+ or F4/80+iNOS+ cells, whereas M2 macrophages were recognized as F4/80+CD206+.
